# Triple Renal Pathology in a Patient With Myeloperoxidase-Associated Vasculitis, Diabetic Nephropathy, and Immunoglobulin A Nephropathy: A Case Report

**DOI:** 10.7759/cureus.116188

**Published:** 2026-09-13

**Authors:** Riddhi Patel, Yash Patel, Supritha Sakhamuri, Akilandanayaki Angamuthu

**Affiliations:** 1 Medicine, Gujarat Cancer Society (GCS) Medical College, Hospital, and Research Centre, Ahmedabad, IND; 2 Medicine, Smt. Nathiba Hargovandas Lakhmichand (NHL) Municipal Medical College, Ahmedabad, IND; 3 Internal Medicine, Guntur Medical College, Guntur, IND; 4 Nephrology, SMC Physicians, Ventnor, USA

**Keywords:** anca-associated vasculitis, diabetic nephropathy, iga nephropathy, mpo-anca, overlap syndrome, plasmapheresis, rapidly progressive glomerulonephritis, renal biopsy, rituximab, sglt2 inhibitors

## Abstract

Rapidly progressive glomerulonephritis (RPGN) is a nephrologic emergency often caused by antineutrophil cytoplasmic antibody (ANCA)-associated vasculitis. While superimposed diabetic nephropathy can alter the clinical baseline, the concurrent presentation of ANCA vasculitis, diabetic nephropathy, and immunoglobulin A (IgA) nephropathy is uncommon. We present a unique case of a triple-overlap renal pathology. A 56-year-old female patient with a history of type 2 diabetes mellitus presented with acute kidney injury (AKI) with a peak serum creatinine of 5.2 mg/dL, accompanied by macroscopic hematuria and nephrotic-range proteinuria (urine protein-to-creatinine ratio >16,000 mg/g). Serological workup revealed highly elevated myeloperoxidase (MPO) antibodies (677 AI). A renal biopsy confirmed three distinct pathologies: focal crescentic glomerulonephritis, nodular diabetic glomerulosclerosis (Class III), and mesangial IgA deposition. The patient received induction therapy with plasma exchange (PLEX), pulse-dose corticosteroids, and intravenous cyclophosphamide. Due to the cumulative toxicity profile of cyclophosphamide, she was transitioned to maintenance rituximab. To address the residual proteinuria driven by the concurrent diabetic and IgA-associated glomerular injury, sodium-glucose cotransporter-2 (SGLT2) inhibitors and angiotensin receptor blockers (ARBs) were added as supportive therapy. Over 14 months, her serum creatinine stabilized at 1.45 mg/dL, MPO titers decreased to 28 AI, and proteinuria improved to ~6,200 mg/g. This case highlights the critical importance of renal biopsy in diabetic patients presenting with atypical renal function decline or active urinary sediment. Furthermore, it demonstrates that a multimodal therapeutic approach is associated with substantial renal recovery in complex overlapping glomerular diseases.

## Introduction

Rapidly progressive glomerulonephritis (RPGN) is a challenging clinical condition characterized by a rapid decline in renal function, often progressing to end-stage renal disease (ESRD) within weeks or months if left untreated. It is typically accompanied by features of nephritic syndrome, including oliguria, hematuria, and proteinuria. Antineutrophil cytoplasmic antibody (ANCA)-associated vasculitis is one of the most frequent etiologies of RPGN in older adults, characterized on histopathology by severe, acute pauci-immune crescentic glomerular injury [1,2].

Clinicians often prematurely attribute deteriorating renal function in patients with long-standing type 2 diabetes mellitus to the natural progression of diabetic nephropathy [3]. However, atypical clinical features - such as the rapid onset of nephrotic-range proteinuria, an abrupt drop in the estimated glomerular filtration rate (eGFR), or the presence of an active urinary sediment containing red blood cells and cellular casts - should serve as critical clinical red flags. These findings warrant a renal biopsy to rule out superimposed non-diabetic kidney disease (NDKD) [4].

While the dual coexistence of diabetic nephropathy with either ANCA vasculitis or immunoglobulin A (IgA) nephropathy (the most common primary glomerulonephritis globally) has been documented, the simultaneous histopathological confluence of all three entities represents an uncommon three-way renal pathologic overlap [5,6]. This convergence creates a significant diagnostic and therapeutic dilemma, as each condition operates through distinct pathophysiological mechanisms. We present the case of a 56-year-old female patient who presented with severe acute kidney injury (AKI) and massive nephrotic-range proteinuria, ultimately revealing a complex triple-overlap pathology that required a highly individualized, multimodal management strategy.

## Case presentation

A 56-year-old female patient with a past medical history of type 2 diabetes mellitus, essential hypertension, and hyperlipidemia was admitted to the hospital for community-acquired pneumonia, which was complicated by severe AKI and macroscopic hematuria. Her baseline serum creatinine prior to admission was 0.9 mg/dL. There was no known family history of kidney disease. Upon admission, her blood pressure was 168/79 mmHg and her weight was 76.4 kg. Her serum creatinine acutely deteriorated to a peak of 5.2 mg/dL, accompanied by massive, nephrotic-range proteinuria and hypoalbuminemia, with a serum albumin level of 2.7 g/dL to 3.2 g/dL. Because this case stems from an acute inpatient consultative encounter, historical outpatient diabetic control metrics - such as the exact duration of her diabetes, an HbA1c drawn prior to this acute illness, and her pre-admission baseline proteinuria - were unavailable in the acute-care medical record.

Diagnostic evaluation

Initial laboratory evaluation demonstrated a severe metabolic acidosis, anemia of chronic disease, and an active urinary sediment containing red blood cells (>30/hpf) and significant proteinuria. To rule out an active infection-related glomerulonephritis triggered by her pneumonia, a comprehensive infectious and structural workup was conducted. A CT of the chest without contrast revealed bilateral multifocal pneumonia and mild small-caliber mediastinal lymphadenopathy. A CT of the abdomen and pelvis without contrast showed no gross hydronephrosis. Sputum cultures grew only normal respiratory flora, and blood cultures showed no growth at five days.

Serological testing revealed highly elevated myeloperoxidase-antineutrophil cytoplasmic antibody (MPO-ANCA) titers at 677 AI. A full serological and electrophoretic panel was obtained to rigorously rule out other competing autoimmune, infectious, and paraprotein-related etiologies (Table 1). Serum protein electrophoresis (SPEP) indicated a pattern suggestive of acute inflammation, and serum immunofixation revealed no monoclonal proteins.

**Table 1:** Comprehensive serologic and diagnostic workup MPO-ANCA: myeloperoxidase-antineutrophil cytoplasmic antibody; PR3-ANCA: proteinase 3-antineutrophil cytoplasmic antibody; AI: antibody index; C3: complement component 3; mg: milligram; dL: deciliter; C4: complement component 4; Anti-GBM IgG: anti-glomerular basement membrane immunoglobulin G; PLA2R: phospholipase A2 receptor; g: gram.

Test	Result	Reference range
MPO-ANCA	677.0 AI (High)	<1.0 AI
PR3-ANCA	<1.0 AI (Negative)	<1.0 AI
Complement C3	158 mg/dL	83-193 mg/dL
Complement C4	24 mg/dL	15-57 mg/dL
Anti-GBM IgG	<1.0 AI (Negative)	<1.0 AI
PLA2R Antibody	<4 (Negative)	<14
Hepatitis B Surface Antigen	Negative	Negative
Hepatitis C Antibody	Negative	Negative
Cryoglobulins	Negative	Negative
Serum Immunofixation	Normal pattern (No monoclonal proteins)	Normal
Free Kappa/Lambda Ratio	1.06	0.26-1.65
Urine Protein/Creatinine Ratio	16,735 mg/g	24-184 mg/g

Given the rapid wane in renal function and active sediment, an urgent renal biopsy was performed (Figure 1).

**Figure 1:**
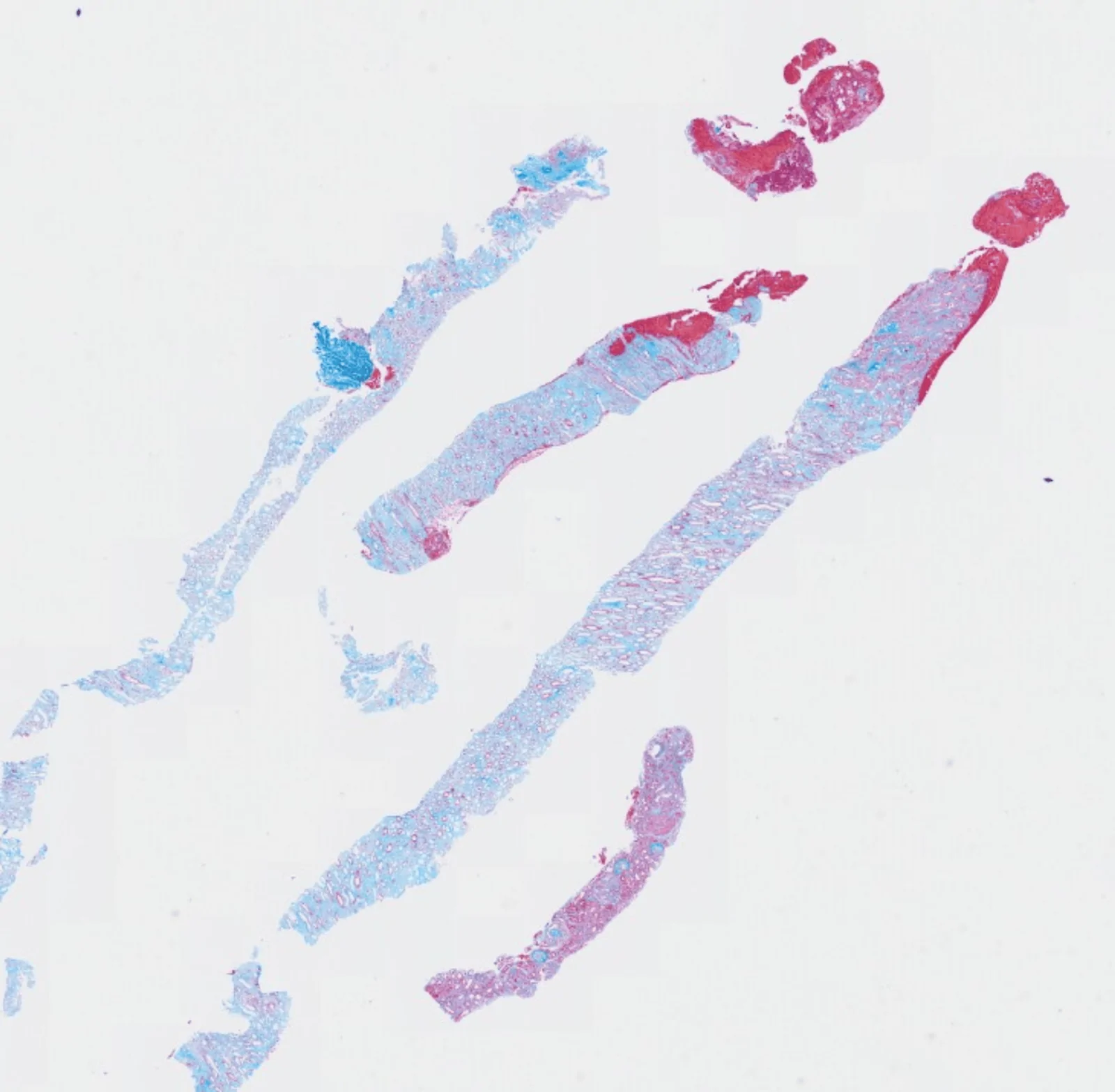
Low-power magnification of the renal biopsy core Light microscopy demonstrating multiple cores of renal cortex and medulla on Masson's trichrome stain (x40).

The histopathology disclosed a striking triple diagnosis. A total of 31 glomeruli were sampled, of which five were globally sclerotic. Segmental sclerosis was absent.

Focal Crescentic Glomerulonephritis

Three of the nine non-sclerotic glomeruli available for light microscopic evaluation demonstrated necrosis or cellular/fibrocellular crescent formation. These findings were consistent with an active MPO-ANCA-associated vasculitis (Figure 2).

**Figure 2:**
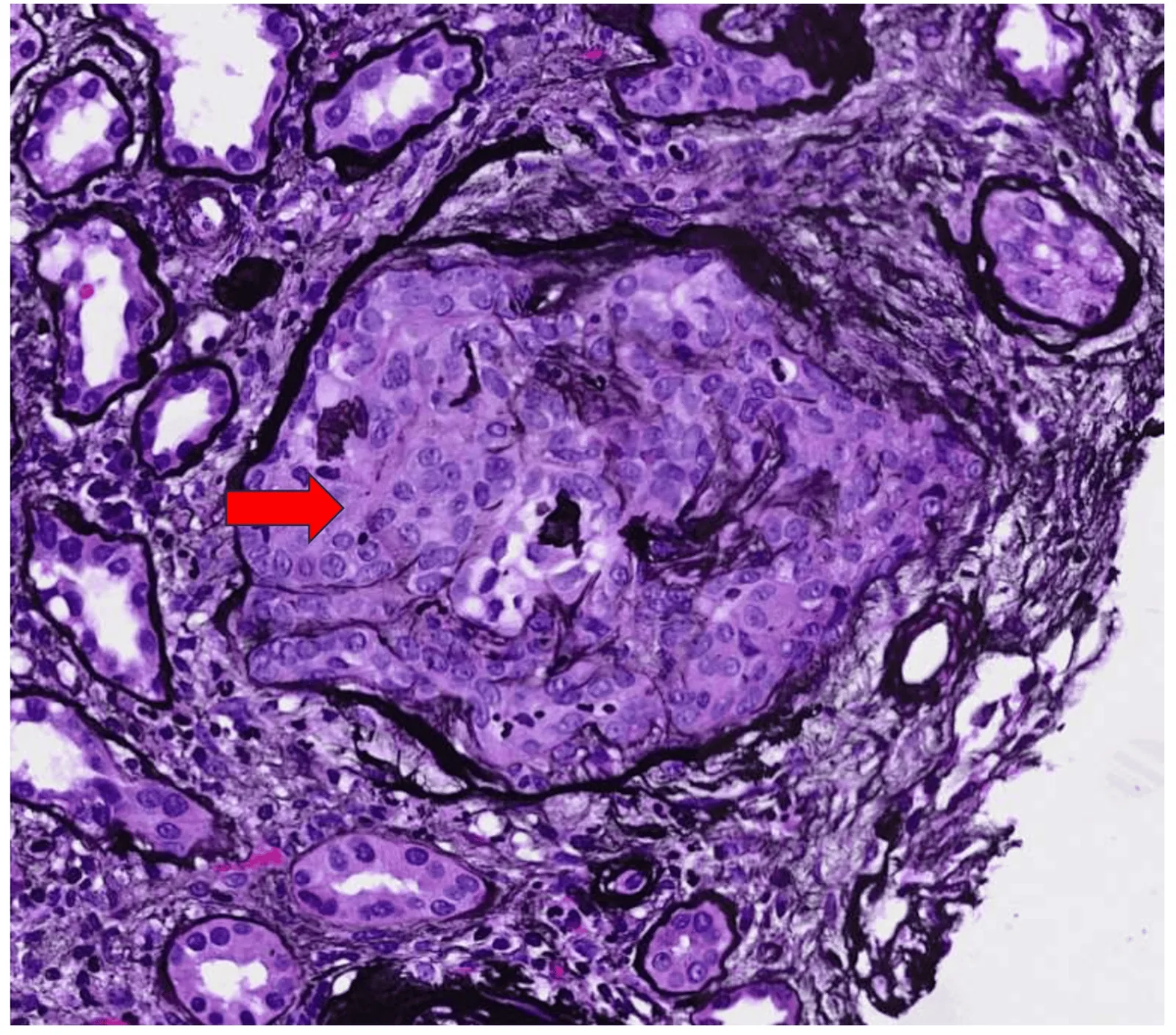
Cellular crescent formation in the glomerulus Light microscopy demonstrating a prominent cellular crescent (indicated by the red arrow) compressing the glomerular tuft, indicative of active antineutrophil cytoplasmic antibody (ANCA)-associated vasculitis on Jones methenamine silver stain (x400).

Nodular Diabetic Glomerulosclerosis (Class III)

This finding established the presence of severe baseline chronic kidney disease (CKD). Light microscopy revealed classic Kimmelstiel-Wilson nodules (Figure 3). The biopsy also demonstrated moderate arteriolar hyalinosis, mild interstitial fibrosis, and mild tubular atrophy (Figure 4). Arterial intimal fibrosis was absent.

**Figure 3:**
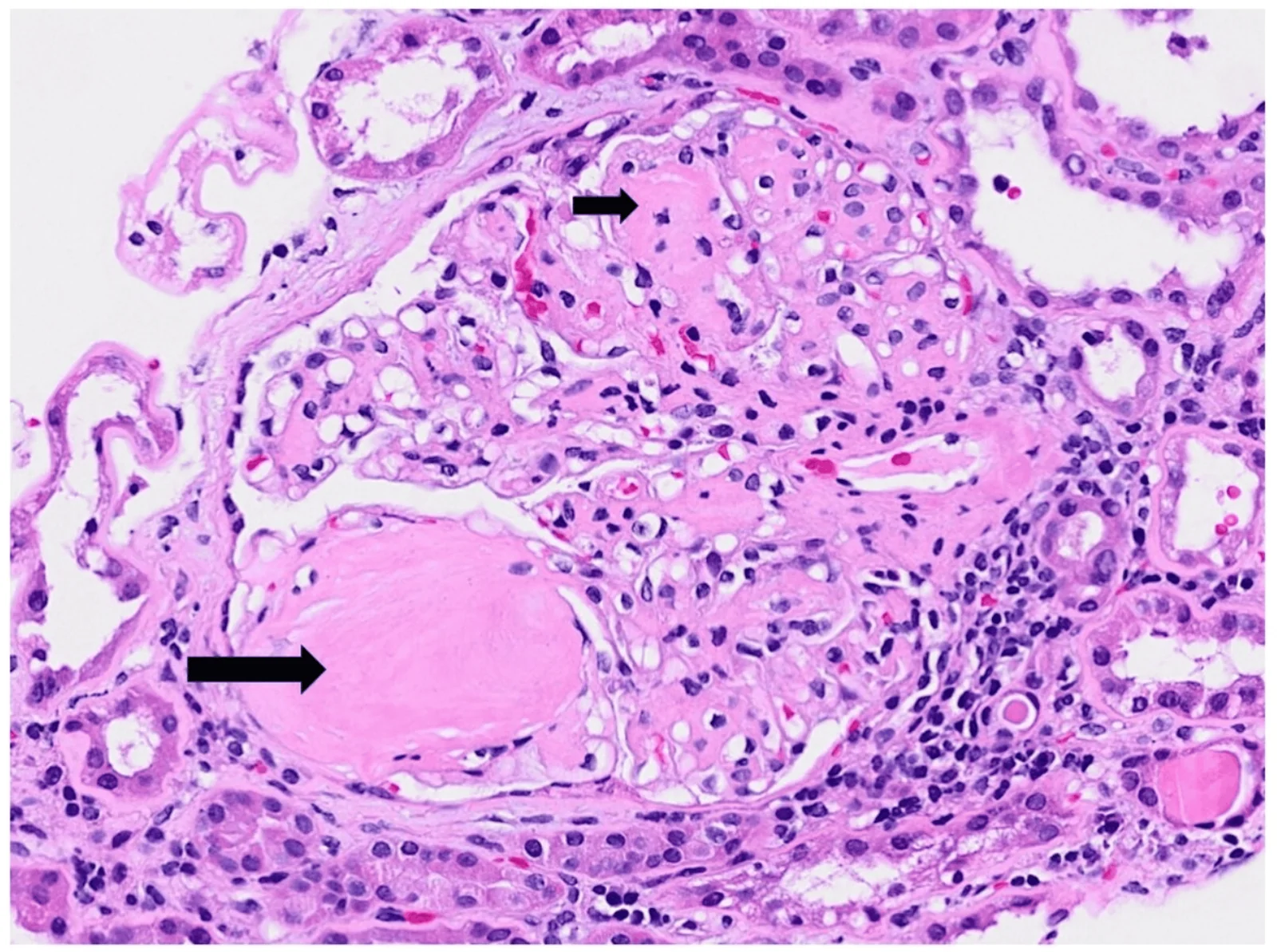
Nodular diabetic glomerulosclerosis Light microscopy revealing nodular glomerulosclerosis with classic Kimmelstiel-Wilson nodules (distinct acellular nodular formations, indicated by black arrows), consistent with Class III diabetic nephropathy on hematoxylin and eosin (H&E) stain (x400).

**Figure 4:**
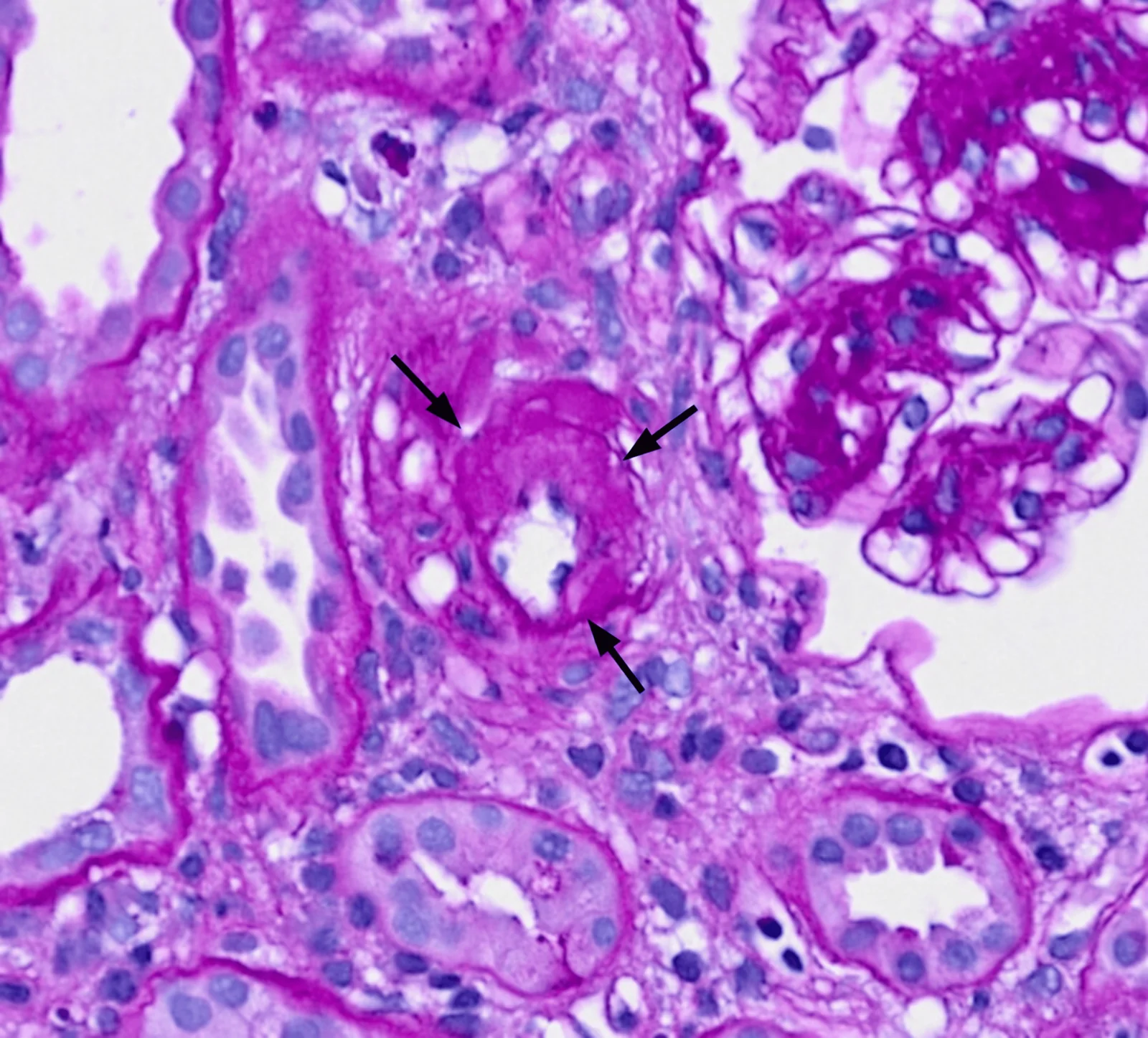
Moderate arteriolar hyalinosis Light microscopy highlighting moderate arteriolar hyalinosis (thickened and eosinophilic arteriolar walls, indicated by black arrows), a classic vascular change characteristic of diabetic kidney disease on periodic acid-Schiff (PAS) stain (x400).

Mesangial IgA Deposition

The third concurrent pathology was confirmed via immunofluorescence, which demonstrated predominantly segmental, granular mesangial staining for IgA (1-2+) (Figure 5) and C3 (1-2+) (Figure 6). Additionally, immunoglobulin G (IgG) was trace to 1+, kappa light chains were 1+, and lambda light chains were trace. The pathologist noted that this degree of mesangial IgA and complement component 3 (C3) deposition was within the upper spectrum of pauci-immune glomerulonephritis. This presentation was deemed most suggestive of an ANCA-associated glomerulonephritis superimposed on diabetic nephropathy and mild background IgA nephropathy. Electron microscopy revealed mesangial matrix expansion containing ill-defined densities, though no definite immune-type electron-dense deposits were identified along the capillary walls. Furthermore, there was moderate to severe epithelial foot process effacement (Figure 7).

**Figure 5:**
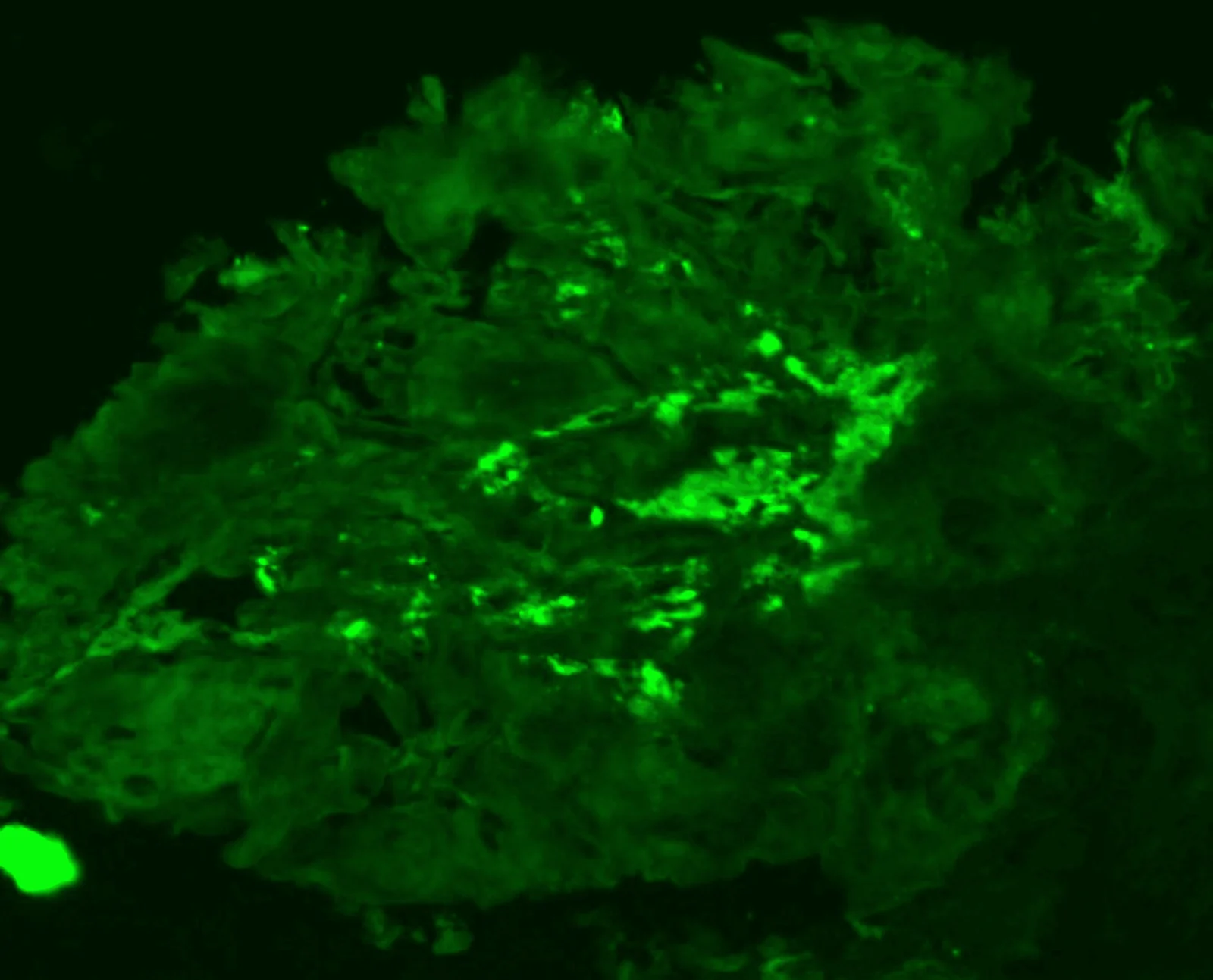
Mesangial immunoglobulin A (IgA) deposition Immunofluorescence microscopy demonstrating bright, granular mesangial positivity for IgA (x400).

**Figure 6:**
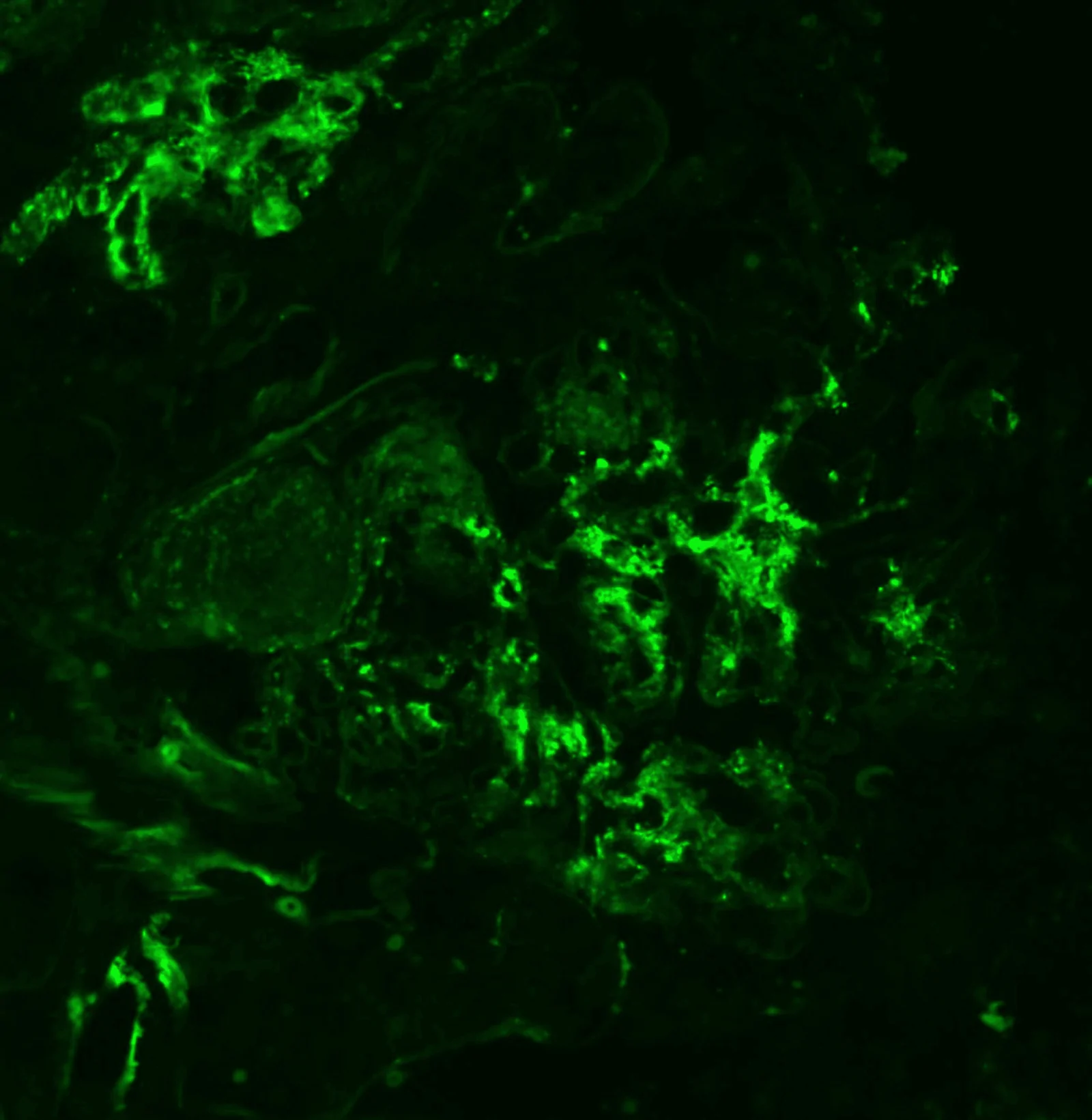
Mesangial complement component 3 (C3) deposition Immunofluorescence microscopy demonstrating bright, granular mesangial positivity for C3 (x400).

**Figure 7:**
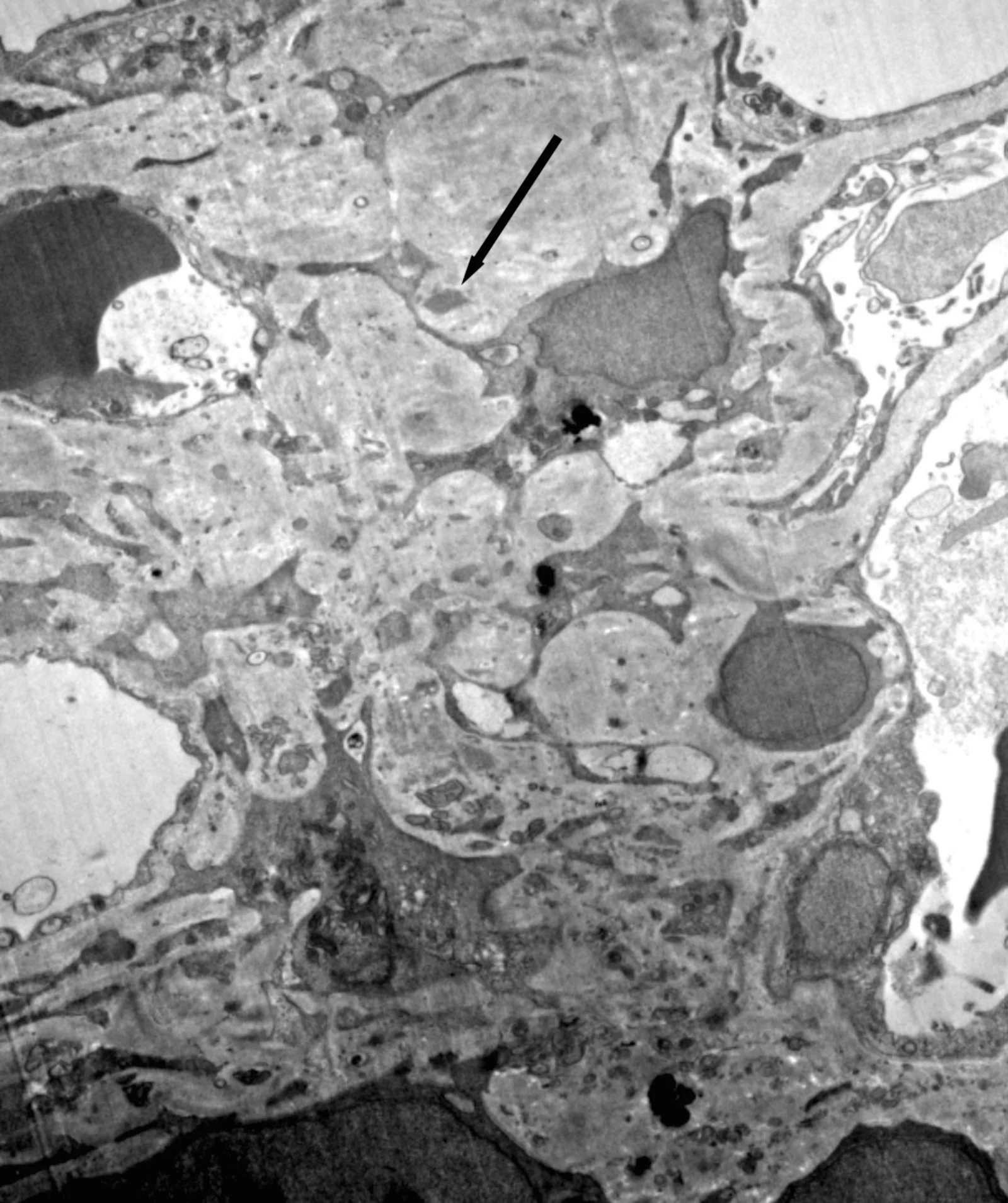
Electron-dense mesangial deposits Electron microscopy revealing small electron-dense deposits localized within the mesangium (indicated by the black arrow), correlating with the immunofluorescence findings of immunoglobulin A (IgA) nephropathy (x8,000).

Therapeutic intervention and clinical timeline

Given the rapidly progressive kidney failure and severe renal dysfunction, plasma exchange (PLEX) was considered as an adjunctive therapy alongside immunosuppression. She completed five sessions of PLEX and received intravenous pulse-dose corticosteroids. She received intravenous cyclophosphamide for induction therapy, initiated with a 750 mg dose.

During the induction phase, she required hospitalization for acute hypoxic respiratory failure, fluctuating AKI, and volume overload. Her regimen was modified following a multidisciplinary discussion with hematology-oncology. Intravenous cyclophosphamide was discontinued in favor of rituximab for maintenance therapy and better tolerability. Maintenance rituximab was administered according to the treating team's individualized protocol, comprising an initial 500 mg dose followed by 1 g every six months. This differed from the MAINRITSAN (Maintenance of Remission Using Rituximab in Systemic ANCA-Associated Vasculitis) trial schedule, which used 500 mg of rituximab on days 0 and 14 and at months 6, 12, and 18 [7]. A timeline of her initial clinical course is detailed in Table 2.

**Table 2:** Clinical timeline Cr: creatinine; MPO-ANCA: myeloperoxidase-antineutrophil cytoplasmic antibody; PLEX: plasma exchange; IV: intravenous; MPO: myeloperoxidase; AI: antibody index; mL: milliliter; SGLT2: sodium-glucose cotransporter-2; mg: milligram; g: gram; dL: deciliter; UPCR: urine protein-to-creatinine ratio.

Timeline	Clinical event and intervention
Day 0	Presentation with community-acquired pneumonia, acute kidney injury (Cr 3.4 mg/dL), and macroscopic hematuria.
Day 1-4	Creatinine peaks at 5.2 mg/dL. MPO-ANCA returns high. Urgent renal biopsy performed.
Day 5-14	Induction immunosuppression: 5 sessions of PLEX, pulse-dose corticosteroids, and first dose of IV cyclophosphamide (750 mg). By day 14, MPO titers decreased to 54.2 AI.
Month 3	Substantial renal recovery. Cyclophosphamide transitioned to rituximab. Oral steroid taper initiated.
Month 11	SGLT2 inhibitor initiated for persistent proteinuria (following a decline from 16,735 mg/g to 7,589 mg/g on immunosuppression alone).
Month 14	Creatinine stable at 1.45 mg/dL. UPCR decreased to 6,226 mg/g.

Chronic management and follow-up

Following the stabilization of the acute vasculitic flare, management shifted toward reducing her hypertension, managing persistent volume overload, controlling severe proteinuria, and slowing the progression of CKD. A follow-up chest radiograph obtained in month 9 demonstrated pulmonary edema with bilateral pleural effusions, reflecting the ongoing clinical challenges of managing volume status in the setting of CKD.

Her pharmacological regimen was optimized to include: (i) Immunosuppression: Maintenance rituximab and a tapering schedule of oral prednisone (tapered down to 5 mg daily). (ii) Renoprotection: An angiotensin II receptor blocker (losartan 50 mg daily) and a sodium-glucose cotransporter-2 (SGLT2) inhibitor (initially dapagliflozin, later transitioned to ertugliflozin 5 mg daily) were introduced to address the proteinuric burden of both her diabetic and IgA-associated glomerular injuries. (iii) Volume control: Loop diuretics (bumetanide 1 mg twice daily) and clonidine.

Over a 14-month follow-up period, her clinical markers demonstrated substantial improvement, as summarized in Table 3. MPO titers steadily decreased, eventually reaching 28 AI at month 11. Her serum creatinine stabilized at a new baseline of approximately 1.45 mg/dL (eGFR 42 mL/min/1.73 m², corresponding to CKD Stage G3b). Her proteinuria, which initially exceeded 16,000 mg/g, decreased to 6,226 mg/g. She remained free of recurrent macroscopic hematuria, with clinically improving lower extremity edema.

**Table 3:** Summary of renal function and serology over time mg: milligram; dL: deciliter; eGFR: estimated glomerular filtration rate; mL: milliliter; min: minute; UPCR: urine protein-to-creatinine ratio; g: gram; MPO: myeloperoxidase; AI: antibody index.

Timepoint	Creatinine (mg/dL)	eGFR (mL/min/1.73 m²)	UPCR (mg/g)	MPO titer (AI)
Admission (Month 0)	5.20	< 15	16,735	677.0
Month 0 (Day 14)	2.60	21	-	54.2
Month 3	1.47	42	9,858	54
Month 6	1.60	38	7,147	-
Month 11	1.52	40	7,589	28
Month 14 (Current)	1.45	42	6,226	28

## Discussion

This case presents an unusual triad of MPO-ANCA vasculitis, diabetic glomerulosclerosis, and mesangial IgA deposition. The presence of NDKD in patients with type 2 diabetes mellitus is reported to range between 10% and 30% depending on biopsy indications and regional practices [4]. Because it is the most common primary glomerulonephritis worldwide, IgA nephropathy is a frequently coinciding condition in this demographic [5]. However, the superimposition of a rapidly destructive, crescentic ANCA-associated vasculitis over dual pre-existing pathologies makes this presentation challenging. Each condition drives renal injury via entirely distinct pathophysiologic mechanisms: long-standing metabolic and hemodynamic strain (diabetic nephropathy), aberrant mucosal immune complex deposition (IgA nephropathy), and acute, autoantibody-mediated necrotizing inflammation (ANCA vasculitis) [2,6].

Diagnostic challenges

A hallmark of advanced nodular diabetic glomerulosclerosis is severe nephrotic-range proteinuria (in this case, peaking at >16,000 mg/g), a condition historically characterized by Kimmelstiel-Wilson nodules and diffuse podocyte effacement [3]. In contrast, isolated ANCA vasculitis conventionally presents with a highly active urinary sediment rich in dysmorphic red blood cells and sub-nephrotic proteinuria [1,2]. Furthermore, infection-associated glomerulonephritis with IgA-dominant deposits was considered due to her initial presentation with pneumonia. However, the absence of active systemic infection (negative blood cultures and normal complementary pathways) and the classic histological findings confirmed the triple primary renal pathologies. The sudden onset of macroscopic hematuria and a sharp plunge in eGFR in this patient served as important clinical signals that prompted the urgent renal biopsy. Failing to recognize that the severe proteinuria and AKI were driven by factors beyond the natural progression of diabetic nephropathy would have precluded the timely initiation of critical immunosuppressive therapy.

Therapeutic challenges

Acute Induction

The acute management of this patient required a delicate and dynamic therapeutic balance. High-dose corticosteroids, which remain essential for inducing remission in severe ANCA vasculitis, inherently worsen glycemic control and foster severe volume retention, both of which were dangerous issues in this diabetic patient presenting with uncontrolled blood glucose and preexisting edema [1].

Moreover, the patient presented with a severely increased serum creatinine of 5.2 mg/dL. The routine use of therapeutic plasmapheresis in ANCA vasculitis has been a subject of debate following the landmark PEXIVAS (Plasma Exchange and Glucocorticoids in Severe ANCA-Associated Vasculitis) trial, which did not demonstrate a long-term benefit in decreasing the composite endpoint of end-stage kidney disease or death for the overall population [8]. Kidney Disease: Improving Global Outcomes (KDIGO) 2024 guidance supports considering adjunctive PLEX when serum creatinine exceeds 3.4 mg/dL (300 μmol/L), when dialysis is required or creatinine is rising rapidly, or when diffuse alveolar hemorrhage is accompanied by hypoxemia [9]. These findings warrant consideration of PLEX rather than mandating its use [9]. In this case, the multimodal induction regimen (including PLEX, pulse-dose corticosteroids, and intravenous cyclophosphamide) was associated with substantial renal recovery, successfully stabilizing her renal function to a CKD Stage G3b baseline [1,10].

Maintenance and Renoprotection

After successful induction, patient management prioritized transition to a long-term, safer maintenance regimen. The patient was transitioned to maintenance rituximab due to the cumulative toxicity profile of prolonged cyclophosphamide. The MAINRITSAN trial data strongly validated this broader strategy by revealing that scheduled rituximab infusions are superior to azathioprine for preventing major relapses [7].

After controlling the acute vasculitis, management focused on the persistent, massive proteinuria caused by concurrent diabetic and IgA-associated glomerular injuries. SGLT2 inhibitors (initially dapagliflozin, later ertugliflozin) were successfully incorporated into her regimen. The major decline in proteinuria - from 16,735 mg/g to 7,589 mg/g - preceded SGLT2 inhibitor initiation, suggesting that immunosuppressive treatment and recovery from the acute vasculitic process accounted for much of the initial improvement. SGLT2 inhibition was subsequently added as supportive renoprotective therapy for the persistent proteinuria. Recent landmark trials, including DAPA-CKD (Dapagliflozin and Prevention of Adverse Outcomes in Chronic Kidney Disease), have demonstrated the profound renoprotective and anti-proteinuric effects of SGLT2 inhibitors, not only in diabetic nephropathy but also specifically in IgA nephropathy subgroups [11,12]. SGLT2 inhibitors provide targeted mechanical relief to chronically damaged glomeruli by reducing hyperfiltration and lowering intraglomerular hypertension [11,12].

Limitations

There are inherent limitations to this single-case observation. Because this case stems from an acute inpatient consultative encounter, historical outpatient diabetic control metrics - such as an HbA1c drawn prior to this acute illness, the exact duration of the patient's diabetes, and her pre-admission baseline proteinuria - are unavailable. Furthermore, because the patient received a multimodal therapy (PLEX, cyclophosphamide, corticosteroids, rituximab, and SGLT2 inhibitors), it is impossible to determine the exact proportional contribution of any single intervention to her renal recovery. It is also challenging to accurately attribute what percentage of her residual proteinuria originates from IgA deposition versus chronic diabetic damage. Lastly, the follow-up period is currently limited to 14 months, requiring ongoing longitudinal observation to determine the long-term viability of this renal preservation strategy.

## Conclusions

The concurrent presentation of MPO-ANCA vasculitis, diabetic nephropathy, and mesangial IgA deposition is an uncommon phenomenon that underlines the diagnostic complexity of NDKD in patients with long-standing diabetes. This case supports the critical clinical necessity to maintain a low threshold for performing renal biopsies in diabetic patients who present with atypical clinical features, such as an active urinary sediment, sudden nephrotic-range proteinuria, or a rapid, unexplained decline in renal function. Moreover, this report demonstrates that successfully managing a complex, triple-overlap glomerular syndrome requires a multiphasic, highly individualized, and multidisciplinary treatment strategy. In the acute setting, severe vasculitic inflammation can respond dramatically to aggressive induction therapies even in patients with significant baseline renal impairment. Once the acute inflammation is controlled, long-term renal preservation needs a synergistic approach: controlling systemic autoimmunity with targeted B-cell depletion while simultaneously reducing intraglomerular hypertension using advanced renoprotective agents. Although the patient maintained persistent severe proteinuria, this dual-targeted approach was associated with substantial recovery from severe AKI and the preservation of kidney function to a stable Stage G3b CKD.
